# Murine Features of Neurogenesis in the Human Hippocampus across the Lifespan from 0 to 100 Years

**DOI:** 10.1371/journal.pone.0008809

**Published:** 2010-01-29

**Authors:** Rolf Knoth, Ilyas Singec, Margarethe Ditter, Georgios Pantazis, Philipp Capetian, Ralf P. Meyer, Volker Horvat, Benedikt Volk, Gerd Kempermann

**Affiliations:** 1 Department of Neuropathology, University of Freiburg, Freiburg, Germany; 2 Burnham Institute for Medical Research, Stem Cell and Regeneration Program, La Jolla, California, United States of America; 3 Laboratory of Molecular Neurosurgery, Department of Stereotactic and Functional Neurosurgery, University of Freiburg, Freiburg, Germany; 4 CRTD – Center for Regenerative Therapies Dresden, Dresden, Germany; Katholieke Universiteit Leuven, Belgium

## Abstract

**Background:**

Essentially all knowledge about adult hippocampal neurogenesis in humans still comes from one seminal study by Eriksson et al. in 1998, although several others have provided suggestive findings. But only little information has been available in how far the situation in animal models would reflect the conditions in the adult and aging human brain. We therefore here mapped numerous features associated with adult neurogenesis in rodents in samples from human hippocampus across the entire lifespan. Such data would not offer proof of adult neurogenesis in humans, because it is based on the assumption that humans and rodents share marker expression patterns in adult neurogenesis. Nevertheless, together the data provide valuable information at least about the presence of markers, for which a link to adult neurogenesis might more reasonably be assumed than for others, in the adult human brain and their change with increasing age.

**Methods and Findings:**

In rodents, doublecortin (DCX) is transiently expressed during adult neurogenesis and within the neurogenic niche of the dentate gyrus can serve as a valuable marker. We validated DCX as marker of granule cell development in fetal human tissue and used DCX expression as seed to examine the dentate gyrus for additional neurogenesis-associated features across the lifespan. We studied 54 individuals and detected DCX expression between birth and 100 years of age. Caveats for post-mortem analyses of human tissues apply but all samples were free of signs of ischemia and activated caspase-3. Fourteen markers related to adult hippocampal neurogenesis in rodents were assessed in DCX-positive cells. Total numbers of DCX expressing cells declined exponentially with increasing age, and co-expression of DCX with the other markers decreased. This argued against a non-specific re-appearance of immature markers in specimen from old brains. Early postnatally all 14 markers were co-expressed in DCX-positive cells. Until 30 to 40 years of age, for example, an overlap of DCX with Ki67, Mcm2, Sox2, Nestin, Prox1, PSA-NCAM, Calretinin, NeuN, and others was detected, and some key markers (Nestin, Sox2, Prox1) remained co-expressed into oldest age.

**Conclusions:**

Our data suggest that in the adult human hippocampus neurogenesis-associated features that have been identified in rodents show patterns, as well as qualitative and quantitative age-related changes, that are similar to the course of adult hippocampal neurogenesis in rodents. Consequently, although further validation as well as the application of independent methodology (e.g. electron microscopy and cell culture work) is desirable, our data will help to devise the framework for specific research on cellular plasticity in the aging human hippocampus.

## Introduction

Adult hippocampal neurogenesis, i.e. the production of new granule cell neurons in the adult hippocampus, has captured the imagination of a wide audience and is beginning to influence hypotheses for clinical medicine. Adult neurogenesis is conserved in all mammalian species studied so far including non-human primates [1], [2], [3], [4], curiously except for most bat species [5]. Detection of newborn granule cells is generally based on the stable incorporation of S-phase marker bromodeoxyuridine (BrdU) into the DNA of a dividing precursor cell and the later immunohistochemical visualization of BrdU in a neuron [6]. Whereas this method is applicable in animal experiments, the detailed description of adult neurogenesis in humans has been limited by the fact that experiments with humans are impossible. The Eriksson study [7] relied on the opportunity that patients had received BrdU for tumor staging purposes within a treatment study. Some of these patients consented to have their brains examined after their death. This rare situation allowed to study adult neurogenesis in humans with the methods established for animals. BrdU incorporation was found in hippocampal granule cells in human individuals as old as 72 years. The Eriksson study was complemented by the discovery of neural precursor cells in surgical specimens from adult human hippocampus [8], [9], [10], [11]. Because of the immense medical implications of adult neurogenesis in humans we intended to find additional information about neuronal development in the adult human dentate gyrus (DG) despite the prevailing limitations and also extended the analysis to the entire lifespan. Several studies have confirmed that adult neurogenesis is present even in the old rodent brain [6], [12], [13] but decreases strongly in early adulthood and remains on a low level thereafter [1], [14], [15], [16].

Adult hippocampal neurogenesis in mice has been described in considerable detail, and distinct developmental stages have been identified [17]. A central phase during this development is associated with the expression of doublecortin (DCX) [18], [19], [20]. This phase ranges from a progenitor cell stage to the calretinin-positive period, during which dendrites and axons of the new cells establish functional connections [21].

DCX is a brain-specific microtubule-associated protein whose exact function is not yet known. It appears to act as microtubule stabilizer in a way that is particularly pertinent to migration [22], [23]. Its presence in the tips of neurites of apparently non-migratory yet immature neurons suggest an additional role in neurite development [24]. Mutation in the human DCX gene causes a characteristic defect in cortical layering, the name-giving doublecortex [25], [26].

DCX can serve as a marker for new neurons in the adult rodent hippocampus as long as the transient nature of its expression is taken into account. In addition, DCX is expressed elsewhere in the rodent (and human) brain, where no link to adult neurogenesis can be made [27], [28], [29]. DCX alone is not specific for adult neurogenesis. Nevertheless, DCX has already been used to detect neurogenesis in the human brain [30], [31] but in those studies, the validity of the marker was only inferred from rodent data and has been questioned [27], [32]. While it is undisputed that true validation for the human hippocampus would require exactly the methods used by Eriksson et al., we reasoned that DCX expression in the DG across the lifespan might be used to generate important information about the combined presence of those parameters in the human dentate gyrus that are known to relate to adult neurogenesis in mice and rats. The underlying assumption is that if the patterns of such features in humans and rodents were highly similar this would also be indicative of a common function. Such functionality can obviously not be proven with the present methodology but even the descriptive part that we can deliver is much more than has been available previously. The assumption itself thus cannot be directly put to test with the approach of this study.

We also made a first attempt to gather semi-quantitative information on how expression of such markers might change across the lifespan as previously described for rodents [6], [12], [16], [33].

## Results

### Doublecortin Expression in the Human Hippocampus

**Figure 1:**
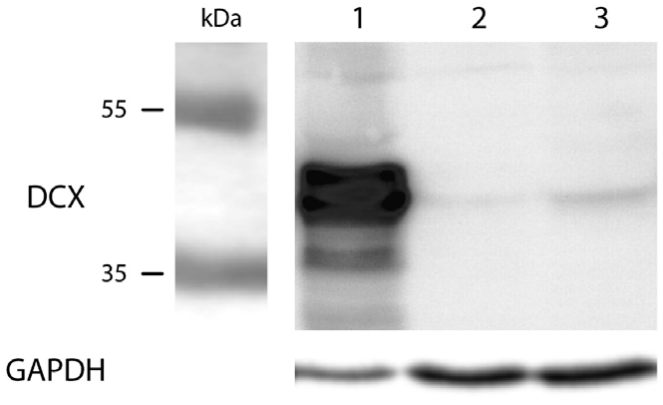
Electrophoretic separation of human brain proteins (25 µg in lane 1, and 50 µg in lanes 2 and 3) and subsequent immunoblotting against DCX and GAPDH. Lane 1: Fetus of GW 20, elective abortion, cortical tissue; lane 2: child of age 3 years, healthy hippocampus; lane 3: adult of age 35 years, suffering from temporal lobe epilepsy (TLE), resected hippocampal tissue. The doublecortin antibody (sc-8066) reacted with proteins in the 45 kDa range, which corresponds to the MG_rel._ of DCX. The signal is strong in the fetal brain and much weaker in the juvenile and adult samples. The GAPDH signal at the 36 kDa position serves as MG_rel_ and loading control.

We performed western blot analyses of normal hippocampal tissue taken from a deceased child and resected hippocampus from a subject suffering from temporal lobe epilepsy with ammonshorn sclerosis (Fig. 1). To test the specificity of our polyclonal antibody fetal cortical brain protein from gestational week 20 (GW20) was run in parallel as positive control. DCX-expression in the fetal brain was much stronger than in the juvenile healthy tissue and the adult epileptic hippocampus. With markers for hypoxia-induced proteins we confirmed that the analysis of our tissue samples was not confounded by perimortal hypoxia (Fig. S1, Table S1). For positive controls of the antibodies applied and negative controls of the immunofluorescence procedure see Fig. S2.

**Figure 2:**
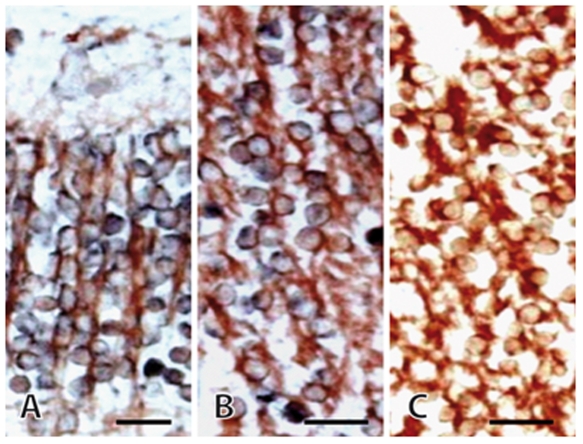
Combined *in situ* hybridization for DCX mRNA (blue-gray) and immunostaining for DCX protein (red) in human fetal tissue (GW 11). A, B, The overlapping staining indicates the co-expression of DCX mRNA and protein. In cortical tissue (A), as well as in the ganglionic eminence (B), the mRNA is not restricted perinuclearly, but is also found in the transverse oriented processes of the round-shaped cells. C, Negative control of the *in situ* hybridization. DCX immunohistochemistry was performed following the *in situ* hybridization protocol with the DCX sense probe. Scale bars, 25 µm.

**Figure 3:**
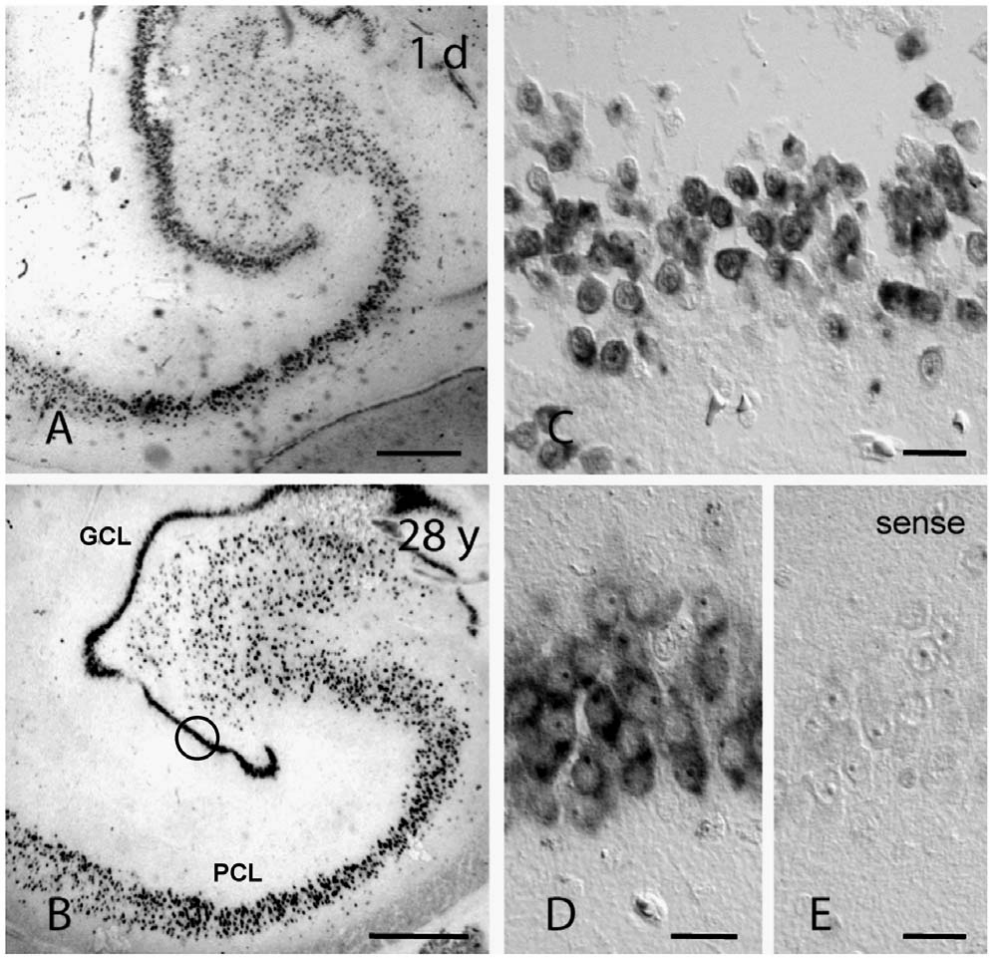
Staining of DCX mRNA by hybridization with an alkaline phosphatase labeled anti-sense cRNA probe in the hippocampus at different ages (non-radioactive *in situ* hybridization). A, B, From birth to adulthood (A: 1 day, B: 28 years of age), hippocampal DCX labeling reveals a distinct staining pattern in the granule cell layer and hippocampus proper. C, In most granule cells (neonatal DG), the mRNA signal occurs outside the nucleus in a small cytoplasmic rim. D, Higher magnification of the DG area marked in (B). E, Application of a labelled sense cRNA probe to a serial section of (B). The absence of sense-probe labelling confirmed the specificity of the detection system for DCX-mRNA/cRNA antisense probe hybrids. Differential Interference Contrast. Scale bars A, B, 1 mm; C-E, 25 µm.

We first examined DCX expression in the developing human DG. At GW11 we found indication of dense DCX expression on the level of both *in situ* hybridization and immunohistochemistry with a complete overlap between both methods (Fig. 2). At birth and 28 years of age *in situ* hybridization for DCX revealed widespread DCX mRNA expression throughout the DG and hippocampus proper (Fig. 3). Persistence of DCX mRNA in the absence of DCX expression, which at 28 years is limited to the putative precursor cells in the SGZ, is consistent with the role of stabilized mRNA in neuronal maintenance [34]. The picture is also similar to the depiction in the Alan Brain Atlas for the murine hippocampus (www.brain-map.org, image series 70946414).

**Figure 4:**
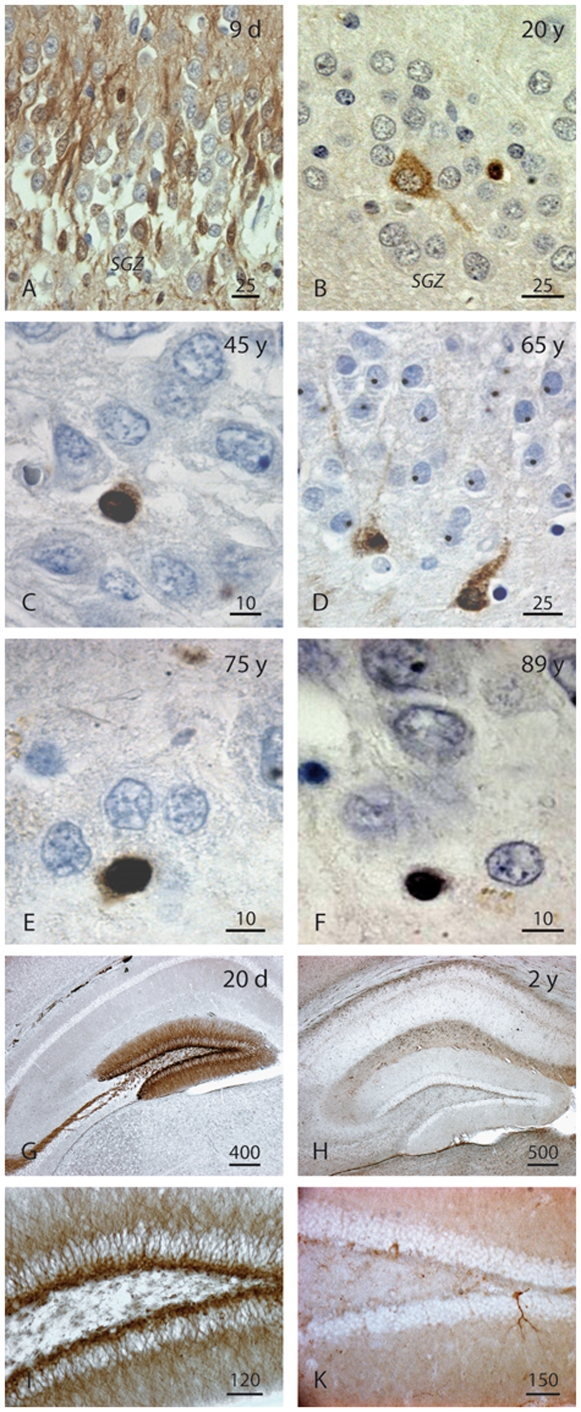
Morphologic variability of DCX+ cells in the granular cell layer (GCL) across the lifespan of humans (A-F) and mice (G-K). Postnatal, numerous DCX+ neurite-bearing cells are scattered throughout the GCL (A). In the adulthood, DCX+ cells are gradually less differentiated in relation to growing age. Initially, some cells show a neuron-like morphology (B,D), whereas the majority of them appears increasingly more undifferentiated and strongly DCX-stained (B-F). In the juvenile mouse hippocampus a multitude of DCX+ cells delineate the GCL formation (G,I). Very old mice, however, exhibit only a few DCX expressing granule cells (H,K). DAB immunohistochemistry of DCX in paraffin embedded brain samples. Length of scale bars as indicated.

**Figure 5:**
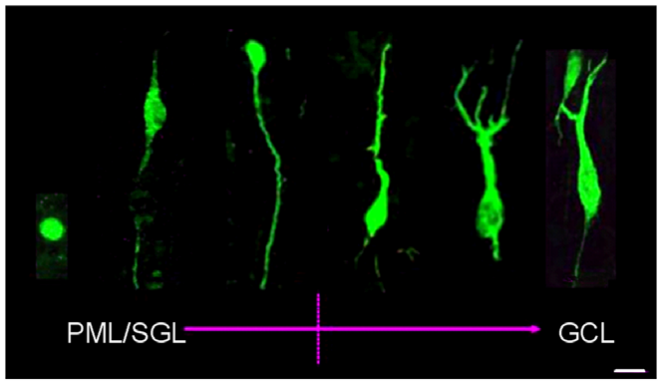
Survey of morphologic variation of DCX-expressing progenitor cells and immature neurons in the DG of a young human subject (5 years-old). Increasing cellular differentiation (dendrites and axons) can be observed starting from the hilar polymorphic layer/subgranular layer (PML/SGL) throughout the GCL up to the inner molecular layer. However, undifferentiated round-shaped cells were always found throughout the entire GCL. This picture is a montage of DCX labeled granule cell and sorted increasingly maturated from left to right. Dendrites are directed towards the granule cell layer (top) and the axons towards the hilus (bottom). Scale bar, 10 µm.

We focused on DCX expression in the DG. DCX-positive (DCX+) cells in the DG could be found in samples across the entire lifespan between 1 day and 100 years of age (exemplary in Fig. 4 and Fig. S3; list of subjects in Table S2). The hilar border of the granule cell layer did not appear as sharp as it is in rodents (see Fig. 4, G-K for comparative images from the mouse brain). No clear subgranular zone (SGZ) could be distinguished (Fig. 4C, D). In young individuals many DCX+ cells showed the dendritic features of immature neurons (Fig. 5), which were not as readily identifiable at older age. However, in both species DCX expressing cells with the morphology of maturing granule cells can be found even at old age, further supporting that no old neurons re-express DCX (Fig. 4D and K). We found co-labeled DCX-positive cells in both male and female specimens but our sample size was to low to address the question of potential gender differences.

### Decreasing Evidence of Neuronal Development in the Aging Human Hippocampus

**Figure 6:**
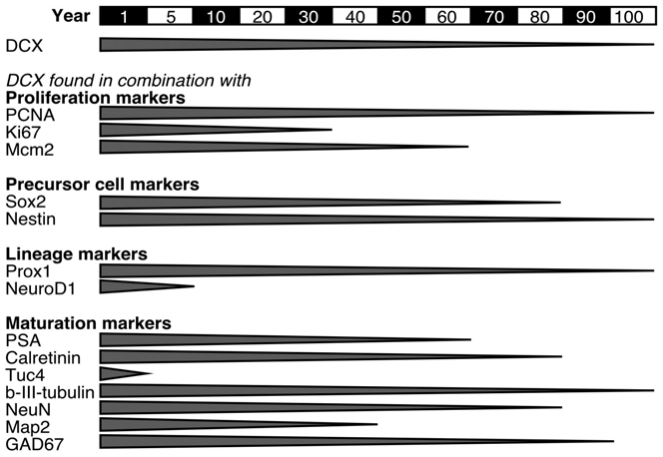
Temporal expression profile and intracellular colabelling in human brain tissue of DCX and additional markers associated with adult hippocampal neurogenesis in rodents.

**Figure 7:**
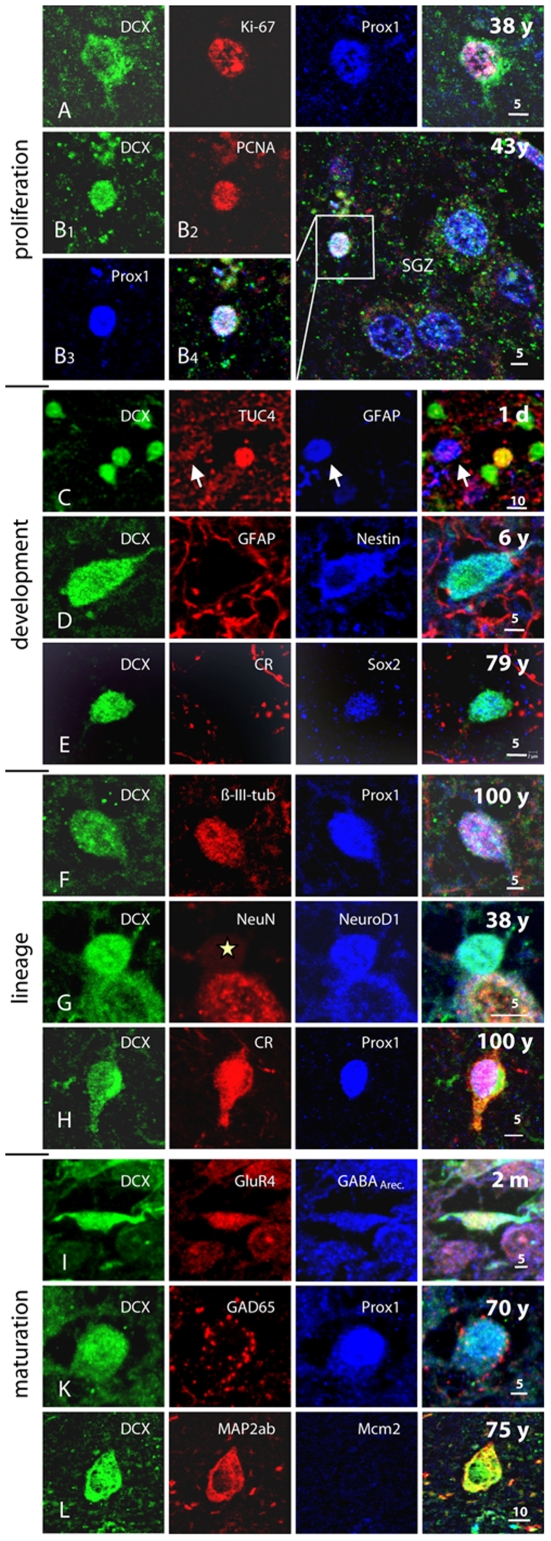
A, B *Proliferation markers* Ki67 or PCNA, co-expressed with the developmental and lineage markers DCX and Prox1, identified SGZ cells as cycling neuronal precursor cells. C–E, The SGZ of juvenile and adult subjects exhibits immature DCX+ cells co-expressing *development markers* characteristic also for developing adult-generated granule cells in rodents. C, In neonates, some immature granule cells co-express DCX and early postmitotic neuronal marker, TUC4. Glia (arrow) did not react against the neuron-specific antibodies applied. D, Early neuro-ectodermal marker nestin labels a bipolar DCX+ cell and neuropil structures not matching GFAP+ astrocytic processes in the PML of a 6 years-old subject. E, Transcription factor Sox2, characteristic for precursor cells, remains expressed in DCX+ cells lacking the transitional maturation marker calretinin. F–H, *Lineage markers* were co-stained with DCX up to oldest age. F, β-III-tubulin, the early neuronal cytoskeleton marker, is expressed also in a DCX+/Prox1+ cell of a 100 year-old individual. G, At age 38 years, NeuroD1, one of the earliest known lineage markers in precursor cells, was detected in a round-shaped DCX+ precursor weakly reactive for maturation marker NeuN (asterisk). H, Even at age 100 years, granule cell-specific transcription factor Prox1 and transient postmitotic neuronal maturation marker calretinin were found together with DCX. I–L, Immunoreactivity for neuronal *maturation markers* in DCX+ cells between 2 month and 75 years. I, Bipolar migratory DCX+ cell expressed markers of neuronal transmission, like glutamatergic and GABAergic receptors (GluR4 and GABA_ARec._) K, DCX+/Prox1+ cells may show GABAergic synapses, here demonstrated by GAD65 in axonal terminals around the cell soma. L, MAP2ab immunostaining could be found among matured DCX+ cells in the adult SGZ. Length of scale bars as indicated.

**Figure 8:**
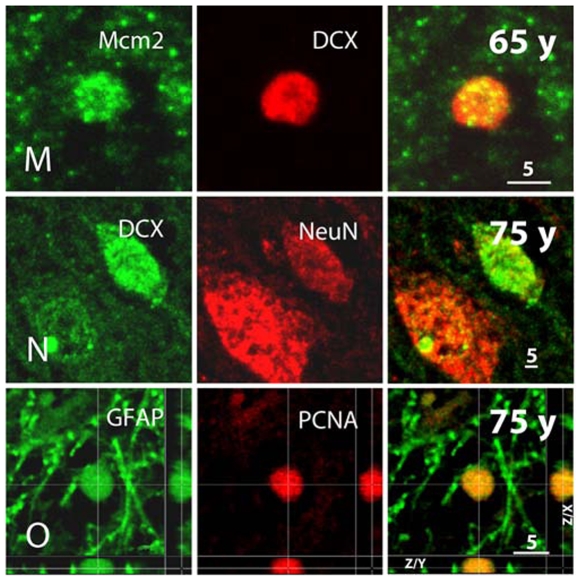
Continuation of Fig. 7. M, DCX and proliferation marker Mcm2, further indicating a proliferative “neuroblast”-like cell. N, Immature neurons express DCX together with postmitotic neuronal marker NeuN; here at age of 75 years. O, Expression of PCNA in GFAP+ cells might be indicative of ongoing stem cell activity in the SGZ but might also reflect classical astrocytic proliferation or DNA repair (see discussion for details). The Z/X- as well as Z/Y-view of the double labeled section clarifies the co-expression of both proteins in the round-shaped cell.

We next established whether DCX expression in the human DG could be linked to other markers that in the rodent are associated with adult hippocampal neurogenesis (Complete list in Table S4). The findings are summarized in Fig. 6, examples are shown in Figs. 7 and 8.

We first examined markers for cell proliferation. PCNA expression in DCX+ cells could be detected at all ages (Fig. 7B). In contrast, we did not detect Ki67 immunoreactivity later than 38 years (Fig. 7A) and Mcm2 later than 65 years (Figs. 6, 7L, 8M). This implies that with advanced age, the level of evidence for ongoing proliferation of DCX positive cells is lower. But in contrast to PCNA (Fig. S1), Mcm2 and Ki67 have not been connected with false-positive signals due to apoptotic cell death [35], [36]. Hence, especially the detection of DCX+/Mcm2+ cells is a relatively strong indicator of persistent proliferation of DCX+ cells (Fig. 8M) — however, in our samples, not beyond the age of 65.

Based on our previous studies in rodents and reports from the literature we next focused on nestin [37], [38], PSA-NCAM [39], NeuroD, Sox2 [40], Prox1 [41], Tuc4 [39], β-III-tubulin, NeuN, and calretinin [21]. These markers could be found in combination with DCX, as predicted from the rodent data [40]. As with the proliferation markers we found that with increasing age fewer of the additional markers showed unambiguous immunoreactivity.

Sox2 is a precursor cell marker that in rodents can be detected in a proportion of hippocampal progenitor cells [42], including a low percentage of DCX+ type-2b and type-3 cells [40]. We found examples of Sox2 in DCX+ cells as late as 79 years of age (Fig.7E).

Prox1 expression, a transcription factor closely related to granule cell development in rodents [43], [44], was found in DCX+ cells across the entire lifespan (Figs. 6 and 7A, F, H). Calretinin, which shows a partial overlap with DCX in the later phase of neuronal development [21] was detected up to the age of 100 years (Fig. 7H). An overlap between DCX and NeuN, characterizing early postmitotic neurons, was found up to 85 years (Figs. 6, 7G, 8N).

Although being glutamatergic neurons, granule cells can co-express GABA and GABA synthesizing enzymes. We detected GAD67/DCX co-localization up to 89 years of age (Fig. 6). GAD65 is primarily expressed in axonal terminals [45]. The fact that the developing new neurons receive early synaptic GABAergic input [46] prompted us to search for evidence of GAD65-positive terminals on DCX+ cells. We found examples such as those presented in Fig. 7K.

### Age-Related Decrease in the Number of DCX-Positive Cells

**Figure 9:**
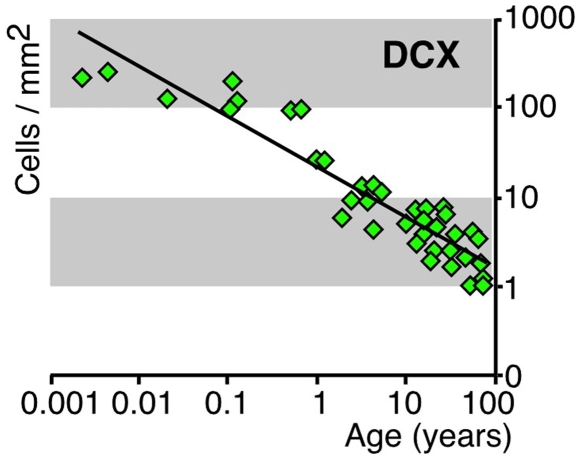
Estimation of DCX+ cell density in the GCL including SGZ across the entire human lifespan. Comprising data from 45 subjects, between 1 day and 94 years, the number of labeled cells in the DG were plotted against the age of the individual. The regression curve shows a log-log-linear decrease (Pearson’s Correlation Coefficient (PPC): -0.95102; *p*  =  0.0001).

Finally, we attempted a semi-quantitative assessment of neurogenesis in the human DG (including the hilar polymorphic layer) across the lifespan. We found that the density of DCX+ cells showed a log-log linear course of the regression curve over the period of life (Fig. 9). Although we found DCX-PCNA double positive cells even at the oldest age (Figs. 6, 7B), about 20% of the PCNA-positive cells showed a co-localization with GFAP (Fig. 8O, Table S3). The nature of the remaining cells could not finally be determined because cell type specific markers are rarely expressed in DNA replicating cells. The PCNA+ cell densities in the DG during life span increase slightly (Fig. S4).

## Discussion

Research on adult hippocampal neurogenesis is often justified by the possible implications that this process might have for human cognition in health and disease. Given this claim the paucity of direct information about adult neurogenesis in the adult and aging human brain is disturbing. We here offer a large data set that provides qualitative and semi-quantitative information about neurogenesis-associated features in the human hippocampus. These data do not offer per se proof of adult neurogenesis in each particular sample but together provide valuable information about the presence of neurogenesis-associated markers in the adult human brain and their change with increasing age.

**Figure 10:**
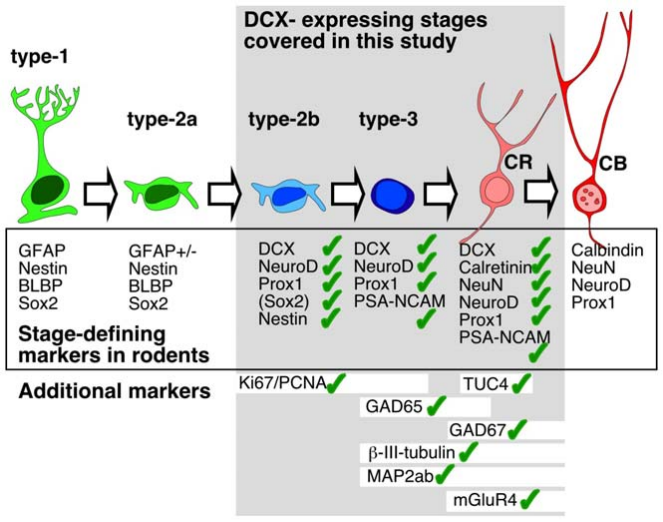
Summary of marker expression in the human hippocampus related to the milestones of adult hippocampal neurogenesis in rodents [17]. The frame indicates the stages of adult neurogenesis covered in this study: all additional markers were tested together with DCX. Marker combinations that we could detect in our samples are marked with a green check. Not all markers were found at all ages, see Figs. 6 for details.

We show that during the first years of life the human DG shows an expression pattern of DCX in numerous combinations with markers of proliferation, precursor cells, maturation, and differentiation that is completely consistent with the information about hippocampal neurogenesis in rodents (Fig. 10). In addition, we found morphological evidence of maturation such as dendrite-like processes (Fig. 5) and hints at the appropriate stage-specific synaptic input (Fig. 7K).

This pattern changes considerable with increasing age but does so in a way that is again more or less consistent with what is known about the age-related changes in adult neurogenesis in rodents (see [47] for review).

### Expression Patterns of Neurogenesis-Associated Markers

Adult hippocampal neurogenesis in mice and rats shows a sequence of marker expressions that has been described in increasing detail [17], [40], [44], [48], [49]. Figure 10 summarizes this pattern for the scope of markers used in this study.

Presence of stem-like cells was suggested by the detection of Sox2 [50], but no radial glia-like cells, the presumed stem cells in the DG of rodents [37], [51] were found. Most studies find that these cells are lacking in the DG of old mice as well, in which neurogenesis is unambiguously detectable. We have postulated that cells with radial glia-like properties but without radial morphology (type-2a), some of which are Sox2+, might act as stem cells in the DG [40]. Again, this remains to be proven, notably for the human brain. But in our sample Sox2 (as well as nestin and Prox1) remained expressed in some DCX-positive cells up to oldest age. Sox2 is expressed in neural stem cells but is not specific to them. Sox2 is also expressed in some astrocytic populations without any known or plausible function in neurogenesis [40].

Because precursor cells are by definition proliferating cells markers of cell division help to substantiate the nature of the DCX-positive cells. Reif et al., who based their conclusions on adult human neurogenesis on immunohistochemistry for proliferation marker Ki67 and studied 60 samples between 25 and 68 years, did not detect positive cells in all cases and found Ki67 up to 62 years ([52] and personal communication). In our sample, Ki67 was not detectable in DCX+ cells beyond the age of 38 and Mcm2 after age 65. On the other hand, the number of dividing DCX+ cells was also extremely low in the aging mouse hippocampus [53], despite ongoing neurogenesis as demonstrated in several studies [54].

In rodents, the phase of DCX expression is also the phase during which cells are eliminated by cell death [55], [56], [57]. There is no indication, however, that DCX itself could be linked to the control of cell death. Quite contrary, the function of DCX is generally seen in maturation processes responding to extrinsic stimuli [23], most notably cell migration and neurite extension, all of which we find in the DCX phase of adult neurogenesis in rodents.

DCX is associated with neurogenesis in the dentate gyrus of adult rodents but expression of DCX is not limited to the context of adult neurogenesis. The overlap with other markers corroborates the idea that the link might exist in the human dentate gyrus as well but in humans as in rodents, DCX is not a neurogenesis marker with high specificity. In the rodent hippocampus its sensitivity is high.

In the murine dentate gyrus DCX shows a nearly complete overlap with the expression of the polysialilated form of the Neural Cell Adhesion Molecule (PSA-NCAM) [19], [39]. We here could confirm that PSA-NCAM can be found immunohistochemically with DAB as chromogen in progenitor cells in the human dentate gyrus (Fig. S3), but PSA-NCAM specific immunofluorescence is considerably more problematic than for DCX. There is, however, a body of literature on PSA-NCAM expression in the adult human brain related to pathology or plasticity [30], [58], [59], [60].To relate these observations directly to adult neurogenesis is as problematic as in the case of relying on DCX alone. The extensive study by Ni Dhuill et al. covering 13 samples from 7 months to 82 years also suggest that besides PSA-NCAM expression in “granule-like cells and their mossy fiber axons” prevailing in young age, other expression sites in the hippocampus become more prominent with increasing age [61]. This raises an important caveat also pertinent to our study, which we have attempted to overcome by co-applying a panel of altogether 24 markers.

Of particular interest are the round-shaped DCX-positive cells, which from their morphology and co-marker expression might relate to the DCX-positive progenitor cells, type-2b and -3 that have been described for the rodent brain. This extrapolation is speculative because further information about the exact course of adult neurogenesis would be needed. Our data might provide at first indication. More detailed analysis is hindered by the fact, however, that these cells are only a subset of all DCX-positive cells and in any given section only few markers can be tested. To describe the course of adult neurogenesis and its dynamics a study based on a cohort of cells that can be followed through the stages of development would be needed. This, however, requires the BrdU-method (or a virus-based approach), which is not readily applicable to humans.

### Caveats

If we take the BrdU-method as the Gold standard (which appears to be generally accepted in the field), there is currently no evidence of adult hippocampal neurogenesis in humans older than 72 years, the oldest sample in the Eriksson study. Because a new BrdU-based study in even older subjects is unlikely to be presented anytime soon, surrogate markers and cumulative supportive evidence gains weight for this age bracket. Single markers will generally not suffice to prove or disprove adult neurogenesis in a particular human sample but contextual information as offered here might help to judge these cases. The use of markers is generally affected by the many caveats associated to investigating human post mortem samples. Combination of markers will decrease the likelihood of false-positive results, but the most serious concern is that with increasing age, markers that in young age are indeed associated with neurogenesis might be increasingly indicative of degeneration and cell death. DNA-synthesis [62] and other cell-cycle-related events have been brought into connection with neuronal cell death in hypoxia and, for example, Alzheimer disease [63]. Also, degenerating cells, more frequently in older brains, might (re-) express immature markers and falsely suggest developmental events. We thus took great care to avoid the detection of false-positive signals due to hypoxic damage or other alterations in relation to the post-mortem interval.

In any case, however, and contrary to the apprehension that with increasing age the amount of non-specific labeling and false-positive marker expression might generally increase, we found a reduction in DCX expression and a reduction in marker overlap. In no case, overlaps not present at a younger age were found in old age. To our knowledge ours it the first study to undertake the analysis of so many histological markers in human samples and this marker loss by itself constitutes an interesting result. Most published studies have been concerned with the age-dependent appearance of histological features, most notably neurofibrillary tangles, senile plaques, deposition of age-pigment, or the increased proliferation of macro- and microglial cells.

Still, the subjects in our series were not healthy but died for extracerebral reasons listed in Table S2. One might reasonably postulate that the data obtained from our samples might rather reflect an underestimate of neurogenesis-associated features. The suggestive presence of DCX-positive cells co-expressing progenitor cell markers even at oldest age might thus indicate that these cells are actually fairly robust against adverse events and the problems associated with the analysis of postmortem tissue.

PCNA is co-factor of DNA polymerases and is expressed during G1 and S phase of the cell cycle. PCNA is critically involved in DNA replication [64] and has thus been established and is widely used as a proliferation marker. Unexpectedly, our semi-quantitative data on PCNA expression did not reveal a substantial change across the age span (but note that the assessment was semi-quantitative and did not obey stereological rules). These data cannot serve as more than an invitation for further studies. They might indicate the increase in astrocytic proliferation in aged brains, although we did not see a substantial overlap with GFAP. PCNA as “proliferation marker” is also problematic in that it might stay on in postmitotic cells [65] and also plays a role in DNA repair [66], [67]. Despite these issues and in the absence of a clear interpretation we did not want to withhold these data.

### Decrease in Neurogenesis-Associated Features with Increasing Age

We also assessed the temporal change in the number of DCX+ cells with age and found an exponential decrease, again similar to the decrease in neurogenesis in rodents [1], [14], [16] and non-human primates [68]. The exponential decrease found in our analysis of DCX-positive cells across the lifespan draws a suggestively similar picture than the related rodent studies. In those, the decrease in adult hippocampal neurogenesis is steepest during the first 3 to 6 months of age and reaches very low and relatively constant levels thereafter [14], [16]. Extrapolated to the human condition and scaled to the human lifespan we might expect that the detectability of neurogenesis-associated features should be highest during adolescence and young adulthood.

In fact we found in the present study that the panel of overlapping markers was exhaustive up to 30 to 40 years (Fig. 6). At that time the overlap comprised Ki67, Mcm2, Sox2, nestin, Prox1, PSA-NCAM, calretinin, and NeuN, essentially the key markers (besides BrdU incorporation), on which most rodent studies are based. This age also coincides with the age suggested by a study using MR spectroscopy to assess adult neurogenesis in the adult human brain [69].

Stereological studies of the human DG, covering samples up to 101 years of age, determined that the number of granule cells in humans is very stable across the lifespan but varies greatly [70], [71]. Given the age-related decline in hippocampal functioning in the absence of dementia, our demonstration of a loss of markers that at least in rodents are related to particular aspects of brain plasticity deserves further analysis. We and others have observed a similar marker loss in the aging rodent hippocampus, although the set of data is far from being conclusive and more studies are needed.

The reduced presence of neurogenesis-associated features in the aged human hippocampus suggests that, as in rodents, neurogenesis is likely to occur on a very low scale, at least if compared to younger age. One of the intriguing questions is thus, how so few new neurons might be functionally beneficial at all. The alternative position to this approach is the theory that because in the course of evolution the ability to produce neurons in adulthood became increasingly restricted, humans are different from animals in that their hippocampus does not rely on this option to alter its neuronal network structure any longer [72].

We have proposed models that help to explain why already very few neurons might make relevant functional contributions in the particular network situations of the hippocampus and we believe that such models also explain why postnatal and young-adult neurogenesis is necessary but increasingly dispensable with increasing age [73], [74]. Also, even absence in oldest age would not argue against a functional role at younger age.

### Conclusions

Overall, our results suggest a pattern of adult neurogenesis in the aging human dentate gyrus that shows large similarities to rodents. Our data alone cannot prove or disprove the true presence or absence of neurogenesis at any age and the consideration of isolated samples might be misleading. Our key results lie in the fact that we see an expression pattern for key markers for adult neurogenesis also in the human brain and that this expression pattern changes with time both qualitatively and quantitatively.

## Material and Methods

Full description of the experimental procedures can be found in the supplemental material (Text S1).

### Specimens

Autopsy cases were selected from the archive at the Department of Neuropathology, University Hospital, Freiburg, Germany, according to age, sex, postmortem interval and lack of clinical or postmortem evidence of neuropathology. Paraffin sections from 3 fetal brain tissue samples (GW11, 20 and 40), hippocampal pieces from a patient suffering from temporal lobe epilepsy and from 51 deceased persons without central nervous damage aged from 1 day to 100 years were analyzed (Table S2).

### Western Blotting and In Situ Hybridization

Western blotting and *in situ* hybridization were performed as described previously [75]. To ascertain the specificity of doublecortin antibody and cRNA anti sense probe in juvenile and adult human hippocampal tissues we included human fetal (GW 11, 20) brain tissue as positive control and reference for western blotting (Fig. 1), *in situ* hybridization (Figs. 2), and RT-PCR (Fig S5).

### Immunohistochemistry

Standard protocols were followed. The detailed description is found in the Supplementary Material. Antibodies are listed Table S5. DCX-immunohistochemistry on mouse tissue was performed as described previously [40]. Negative controls were performed by omitting primary antibodies (see Fig. S2C). They did not showed any fluorescence signals. DCX-immunohistochemistry on mouse tissue was performed as described previously [57].

## Supporting Information

Text S1 Supplemental material and methods.
(0.06 MB DOC)

Figure S1 Test of juvenile and adult human GCL specimens for potential perimortal hypoxia. A, No co-expression of DCX with the Heat Shock Protein 27 (HSP27) was detected and no signs of microglia activation (CD68) were found in the samples. B, Similarly, microglial marker Glut1 and Hypoxia-Induced Factor 1α (HIF-1α) were not increased. C, Matrix MetalloProteinases (MMP) are important for migration of young cells within established neural networks. Here, MMP9 shows strong co-labeling with DCX, whereas the same cell is not stimulated to express Vascular Endothelial Growth Factor A (VEGF-A), which would be indicative of a hypoxic milieu. D, Proliferation markers like PCNA might show expression in cells undergoing apoptosis. Here, a DCX+ cell in a 2 month-old GCL presents signs of programmed cell death by activated-Caspase-3 (Casp-3). E, Whereas apoptosis is very physiologic in neonatal age and crucial for neuronal network consolidation, the DG of a 58 years-old subject revealed no signs of degradation. The lack of PCNA- and activated caspase-3 labeling in the presented cell argues against the interpretation that DCX expression in higher age is induced by stress factors. Scale bars, 5 µm and 10 µm as indicated.
(0.33 MB JPG)

Figure S2 A, B, Proof of reactivities of anti activated-caspase-3 (A) and anti heat-shock protein HSP27 antibodies (B) in a glioblastoma multiforme specimen. Positive cells are devoid of DCX and PCNA immunoreactions, resp. C, Technical control incubation: Omitting primary antibodies, the incubation of serial sections with secondary antibodies alone failed to generate any specific fluorescence signal. D, E, Search for DCX expressing neuronal cells outside the hippocampus. Bipolar DCX+ cells could be detected in the parietal neocortical parenchyma (LII) (D) as well as in the temporal migratory stream (TMS) of the piriform Cortex/Amygdala (E). There are neither co-labelings with the microglial marker CD68 and the heat-shock-protein HSP27 nor with the cell type specific markers NeuN and GFAP. Scale bar length as indicated.
(56.30 MB TIF)

Figure S3 PSA-NCAM expresssion. PSA-NCAM is expressed in a small hippocampal SGZ cell of a 68 years-old male. DAB staining.
(0.60 MB JPG)

Figure S4 Estimation of PCNA+ cell densities in the DG across the entire human lifespan. Comprising data from 25 patients, aged between 1 day and 100 years (see Table S1), the number of labeled cells in the GCL were plotted against the age of the individual. The regression curve enables a log-log-linear interpretation. PCNA+ cell densities increase slightly with age (PPC: 0.154; p = 0.463).
(0.10 MB TIF)

Figure S5 Doublecortin mRNA. Generation, purification and cloning of human DCX-specific cDNA inserts aimed to generate DIG-labeled cRNA probes for northern blotting and in situ hybridization. A, After bulk separation in a preparative 1.5% agarose gel, the fetal human brain DCX-PCR product (510 bp, lane 2) was recovered and in B, ligated into a pSPT19 vector (3.604 bp, lane 1), cloned and approved for correct insertion by digestion with EcoRI and HindIII resulting in a linearized vector (3.104 bp) and the DCX insert (lane 2) designed for the cRNA-DIG probe generation by in vitro transcription. C, Separation of total RNA from fetal brain in an ethidium bromide stained acrylamide gel (lane 1) showing strong fluorescence of the 18S and 28S ribosomal rRNAs. D, After blotting on nylon membrane the immobilized RNA was simultaneously hybridized with the DIG-labeled antisense probes against DCX and β-actin mRNAs and processed for visualization of the mRNA/cRNA-DIG binding sites by the enhanced chemiluminescence method. Besides the very strong signal of β-actin mRNA serving as housekeeping transcript and loading control a faint band of DCX mRNA was visible at the correct position of 9.54 kb.
(0.26 MB TIF)

Table S1 Analysis of perimortal hypoxic changes.
(0.04 MB DOC)

Table S2 Data of subjects included in the study.
(0.13 MB DOC)

Table S3 Quantification of PCNA and GFAP co-expressing undifferentiated cells in the dentate gyrus.
(0.05 MB DOC)

Table S4 List of primary antibodies.
(0.10 MB DOC)

Table S5 Fluorochrome conjugated secondary antibodies and optical/technical parameters for their detection in the Leica TCS NT confocal laser scanning microscope.
(0.03 MB DOC)
