# Supplementary material for: Murine Features of Neurogenesis in the Human Hippocampus across the Lifespan from 0 to 100 Years
Source: PLoS One. 2010 Jan 29;5(1):e8809. doi: 10.1371/journal.pone.0008809 (PMC2813284; doi:10.1371/journal.pone.0008809)
Supplement: Text S1 — Supplemental material and methods. (0.06 MB DOC) [file pone.0008809.s001.doc]

**Text S1**

**Supplemental Material & Methods**

***Immunohistochemistry***

*Tissue processing*

Representative formalin-fixed and paraffin-embedded autopsy tissues were routinely cut at 5µm, applied to electrostatically modified “SuperfrostPlus” slides (Langenbrinck, Teningen, Germany), heated at 80°C for 1 h, dewaxed with xylene, rehydrated, and treated in citrate buffer, pH 6.0, for 4 min in a pressure cooker to demask antigenic sites. Following washes in PBS, the slides are ready for light microscopy as well as immunohistochemistry.

*Antibodies and fluorochromes*

The primary antibodies, their characteristics, suppliers, and working dilutions are summarized in Table S4. Fluorochrome conjugated secondary antibodies were listed in Table S5 including corresponding optical properties and parameters for detection in the confocal laser scanning microscope.

*Single immunoperoxidase labeling*

Immunoperoxidase labeling was performed following recommendations of the Vector ABC kit manufacturer (Vector Laboratories, Burlingame, CA). The rehydrated and H_2_O_2_ treated specimens were incubated with blocking buffer (2% rabbit normal serum in PBS/0,1% Triton X-100) for 1 h. Primary antibodies were diluted in a 1% blocking buffer and applied to sections mounted in “Shandon Coverplate” trays (Thermo Electron Corp., Pittsburgh, PA) overnight at room temperature. The next day incubation steps were performed with biotinylated rabbit anti-goat IgG for 2 h following the ABC reagent for 4 h. The peroxidase label was visualized with diaminobenzidine as chromogen. Slices were dehydrated and cover slipped in “Histofluid” (Marienfeld, Germany). Control incubations performed without primary and/or secondary antibodies revealed no specific staining.

*Multicolor fluorescence labeling and confocal microscopy*

Paraffin sections treated and mounted as described above were blocked with 5% donkey normal serum in PBS/0,1% Triton X-100 for 30 min and incubated overnight in a mixture of primary antibodies of different species diluted in 1% blocking buffer. After thoroughly rinsing, the secondary antibodies were allowed to react for 4 h. For quenching autofluorescence of tissue and lipofuscin, the specimen were equilibrated in a 50% PBS/ethanol mixture for 2 min followed by sudan B (0,1%) dissolved in 70% ethanol [1]. After thoroughly washings with 50% PBS/ethanol and PBS the sections were coverslipped in the anti-fading agent “Mowiol” (Calbiochem, La Jolla, CA) and stored overnight at 4°C. Immunofluorescences were scanned at 1024 x 1024 pixel resolution with a confocal laser scanning microscope (Leica TCS NT, Heidelberg, Germany) equipped with an argon/krypton laser and filter settings shown in Table S5. Three channel recordings were performed one after another in Z-series with gaps of 250 nm in between.

*Digital illustrations*

Light micrographs of immunoperoxidase labeled specimens were taken on the Leica DMRX microscope equipped with a digital camera DC300F (Leica Microsystems, Heidelberg, Germany) and Leica Image Manager IM1000 software. Confocal stacks of Z-planes were stored as TIF-files and processed in the Imaris 4.2 software (Bitplane AG, Zurich, Switzerland) for projection and orthogonal reconstruction of Y/Z and X/Z views. Compiling was carried out by means of the Adobe Creative Suite 2 software. Murine specimens were analyzed on a Leica TCS SP2 as described previously [2].

***Western immunoblotting***

Human tissue samples were taken (i) from fetal brain after elective abortion at GW20, (ii) from snap-frozen resectioned hippocampal specimens of patients with ammonshorn sclerosis (AHS) suffering from temporal lobe epilepsy and (iii) from a deceased child without known (brain) pathology and histopathological alterations of brain tissue (for peculiarities of individual specimens, see Table S2). SDS-PAGE and immunoblotting were done in detail according to our previously described protocol [3]. The native fetal cortical and postnatal hippocampal tissues were homogenized by sonification in sample buffer, electrophoresed, and blotted on Immobilon PVDF-membranes. For detection of DCX protein, the polyclonal goat anti-DCX antibody (sc-8066, Santa Cruz Biotechnology, Santa Cruz, CA) was applied and detected with the Enhanced Chemiluminescence Detection System (ECL, Amersham Pharmacia Biotech, Freiburg, Germany). As loading control subsequently we performed an incubation of the PVDF membrane with mouse anti GAPDH (Chemicon MAB374) and visualized the signals in the same manner.

***Molecular biology***

*RNA isolation, primer design, RT-polymerase chain reaction*

Generally, we followed the procedure previously described except for primer sequences and PCR-temperature regime [4]. Total RNA was extracted from human fetal brain (GW 11) with the peqGold RNA Pure system following the instruction manual (peqLab Biotechnologie, Erlangen, Germany). A primer pair of 22 nucleotides each was designed from the human DCX CDS sequence (NCBI GenBank Acc: NM_178153). The following oligonucleotides were synthesized: 5´-GCAATGGGGACCGCTACTTCAA (forward primer) spanning the nucleotides 239-260 and 5`-GACAACCCCGGTCTCCAGTTTG (reverse primer) spanning the nucleotides 717-738. Linker sequences with EcoRI and HindIII restriction sites were added enabling later insertion of the PCR product into the pSPT19 plasmid. The primer pair allowed for the specific amplification of a 500 bp cDNA. Conditions for PCR procedure are the followings: Cycle number: 30, initial denaturation step: 94°C, 3 min. Each cycle consisted of a denaturation step at 94°C (45 sec), an annealing step (56°C, 1 min) and an elongation step (72°C, 1 min) with a final 10 min elongation after the last cycle. The PCR product was separated on and recovered from the agarose gel, digested with EcoRI and HindIII, ligated into EcoRI and HindIII treated pSPT19 plasmids, cloned and propagated in host E.coli strain DH5α, and finally sequenced. It was proved to be 100% identical to the master CDS sequence published and demonstrated exclusive specificity against the intended target gene.

*cRNA probe preparation and northern blotting*

We followed the protocol of Singec et al. (2003) (4) to synthesize DIG-labeled sense and antisense DCX cRNA probes using the “SP6/T7-Polymerase Digoxigenin (DIG) Labeling and Transcription Kit” (Roche Diagnostics, Mannheim, Germany) [4]. The sense DCX cRNA probe was generated by SP6 polymerase from the pSPT19 plasmid linearized with HindIII, the antisense probe was generated by T7 polymerase from pSPT19 plasmid linearized with EcoRI. Total RNA from a human fetal (GW 11) brain was separated, blotted onto a positively charged Nylon membrane (Roche Diagnostics, Mannheim, Germany) and hybridized simultaneously with DIG-labeled antisense probes against DCX- and β-actin poly(A)^+^ RNAs and processed for visualization of the mRNA/cRNA-DIG hybrids using the ECL method.

*Non-radioactive in situ hybridization*

Paraffin sections (5µm) mounted on Superfrost Plus slides were dewaxed, rehydrated, and postfixed in 4% PFA in PBS for 20 min. After washing the specimens were subjected to a modified labeling protocol published by Singec and colleagues [4]. Hybridization was carried out overnight at 59°C with gentle agitation. For immunohistochemical detection of the DIG-labeled DCX cRNA probes we applied the DIG Nucleic Acid Detection Kit (Roche Diagnostics) with alkaline phosphatase conjugated anti-DIG antibodies and NBT/BCIP as chromogen. The development of the blue-gray stain was accomplished after 4 h at room temperature in the dark. Five repeats of this protocol with hippocampal tissues from differently aged subjects revealed corresponding results.

*Double labeling (in situ hybridization followed by immunohistochemistry)*

After *in situ* labeling of DCX mRNA (see above) some stained slides were taken for subsequent immunocytochemical detection of DCX protein in the same tissue slice. The immunohistochemistry was performed as mentioned above using the ABC kit (Vector Lab) and AEC as red chromogen instead of DAB. Importantly, regarding the solubility of the ISH and IHC labels, the slices were not allowed to dehydrate in ethanol. They were coverslipped in Kaiser´s glycerol gelatine (Merck, Darmstadt, Germany).

***Estimation of the density of DCX expressing cells and correlation with age***

Ten paraffin sections each of 33 patient’s anterior hippocampal specimens (see Table S2) were prepared. The first, fifth and tenth section of each series has been immunolabeled against DCX using the ABC protocol and diaminobenzidine as described. Therefore, from each hippocampus we analyzed three sections at non-overlapping Z-planes. PCNA labeled specimens from 26 subjects were identical analyzed and correspondingly estimated the number/density of PCNA/GFAP double labeled cells (Table S3). Micrographs using the Leica PL Fluotar 1.6x/0.05 objective were digitally captured with the digital camera DC300F of the Leica DMRX microscope and evaluated with the Leica Image Manager IM1000 software. The GCL including the SGZ was outlined and the area estimated as mm². Using bright field optics and the immersion objective (Leica PL Apo 40x/1.255-0.75 OIL) all immunolabeled cells within the marked area were counted by three observers blindly with respect to the case ID number. The numbers of labeled cells per mm² were calculated and the average for the three sections of each patient’s hippocampus estimated.

Sample values were analyzed with the SAS 8.2 software. The data allowed a log-log-linear interpretation of the correlation between the parameters: age and cell density with a very high significance for the DCX+ cells (Pearson’s correlation coefficient: -0.95102; p=0.0001) and a non significant, but tendentious increase for the PCNA+ cells (PCC: 0,154, p=0.463)

**References**

1. Romijn HJ, van Uum JF, Emmering J, Goncharuk V, Buijs RM (1999) Colocalization of VIP with AVP in neurons of the human paraventricular, supraoptic and suprachiasmatic nucleus. Brain Res 832: 47-53.

2. Steiner B, Klempin F, Wang L, Kott M, Kettenmann H, et al. (2006) Type-2 cells as link between glial and neuronal lineage in adult hippocampal neurogenesis. Glia 54: 805-814.

3. Singec I, Knoth R, Ditter M, Hagemeyer CE, Rosenbrock H, et al. (2002) Synaptic vesicle protein synaptoporin is differently expressed by subpopulations of mouse hippocampal neurons. J Comp Neurol 452: 139-153.

4. Singec I, Knoth R, Ditter M, Frotscher M, Volk B (2003) Neurogranin expression by cerebellar neurons in rodents and non-human primates. J Comp Neurol 459: 278-289.
