# Supplementary figures and images for: Murine Features of Neurogenesis in the Human Hippocampus across the Lifespan from 0 to 100 Years
Source: PLoS One. 2010 Jan 29;5(1):e8809. doi: 10.1371/journal.pone.0008809 (PMC2813284; doi:10.1371/journal.pone.0008809)

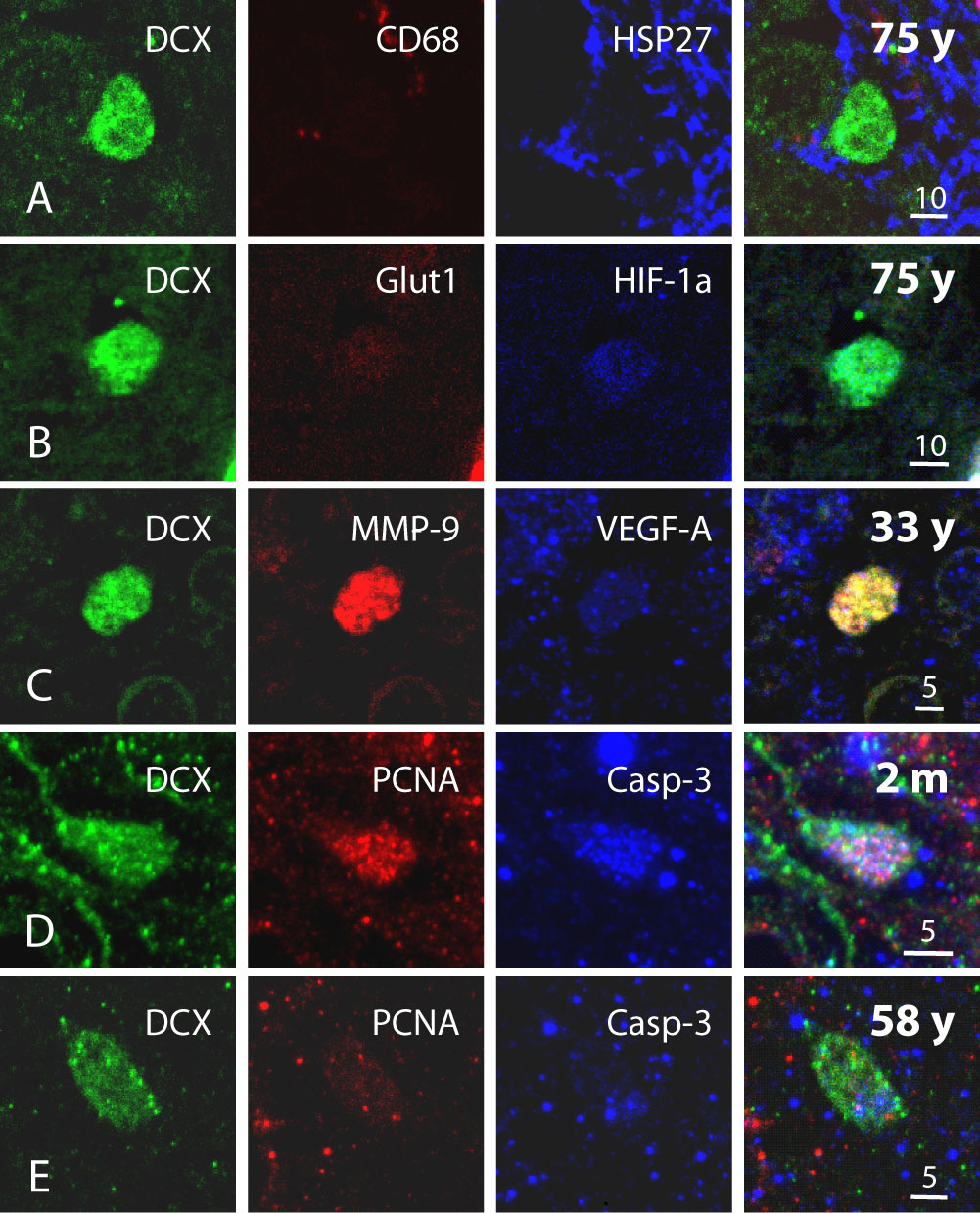

Supplement: Figure S1 — Test of juvenile and adult human GCL specimens for potential perimortal hypoxia. A, No co-expression of DCX with the Heat Shock Protein 27 (HSP27) was detected and no signs of microglia activation (CD68) were found in the samples. B, Similarly, microglial marker Glut1 and Hypoxia-Induced Factor 1α (HIF-1α) were not increased. C, Matrix MetalloProteinases (MMP) are important for migration of young cells within established neural networks. Here, MMP9 shows strong co-labeling with DCX, whereas the same cell is not stimulated to express Vascular Endothelial Growth Factor A (VEGF-A), which would be indicative of a hypoxic milieu. D, Proliferation markers like PCNA might show expression in cells undergoing apoptosis. Here, a DCX+ cell in a 2 month-old GCL presents signs of programmed cell death by activated-Caspase-3 (Casp-3). E, Whereas apoptosis is very physiologic in neonatal age and crucial for neuronal network consolidation, the DG of a 58 years-old subject revealed no signs of degradation. The lack of PCNA- and activated caspase-3 labeling in the presented cell argues against the interpretation that DCX expression in higher age is induced by stress factors. Scale bars, 5 µm and 10 µm as indicated. (0.33 MB JPG) [file pone.0008809.s002.jpg]

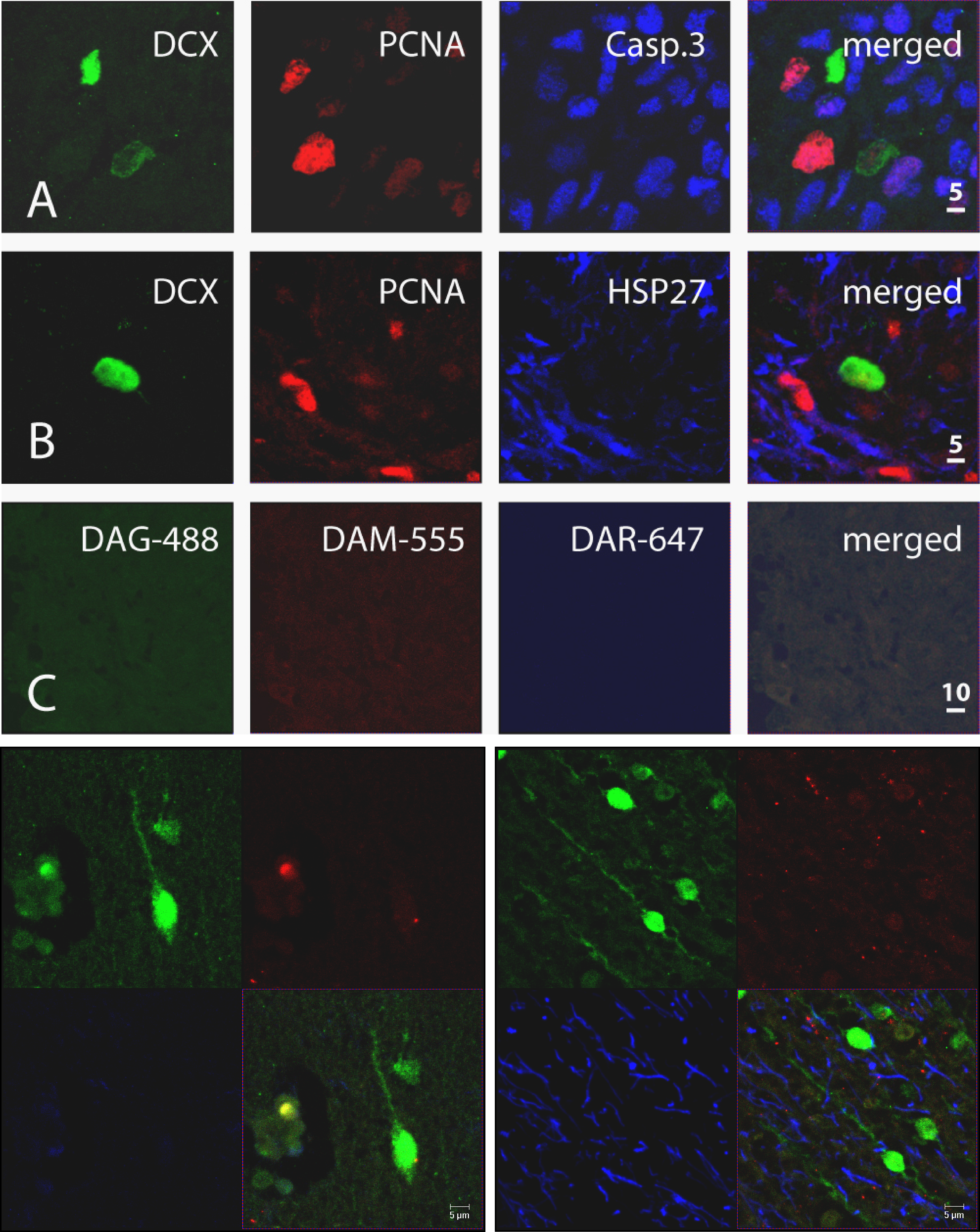

Supplement: Figure S2 — A, B, Proof of reactivities of anti activated-caspase-3 (A) and anti heat-shock protein HSP27 antibodies (B) in a glioblastoma multiforme specimen. Positive cells are devoid of DCX and PCNA immunoreactions, resp. C, Technical control incubation: Omitting primary antibodies, the incubation of serial sections with secondary antibodies alone failed to generate any specific fluorescence signal. D, E, Search for DCX expressing neuronal cells outside the hippocampus. Bipolar DCX+ cells could be detected in the parietal neocortical parenchyma (LII) (D) as well as in the temporal migratory stream (TMS) of the piriform Cortex/Amygdala (E). There are neither co-labelings with the microglial marker CD68 and the heat-shock-protein HSP27 nor with the cell type specific markers NeuN and GFAP. Scale bar length as indicated. (56.30 MB TIF) [file pone.0008809.s003.tif]

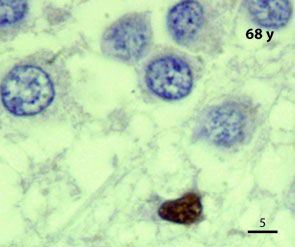

Supplement: Figure S3 — PSA-NCAM expresssion. PSA-NCAM is expressed in a small hippocampal SGZ cell of a 68 years-old male. DAB staining. (0.60 MB JPG) [file pone.0008809.s004.jpg]

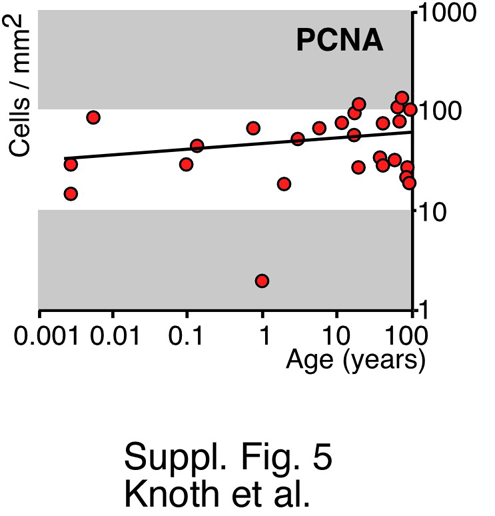

Supplement: Figure S4 — Estimation of PCNA+ cell densities in the DG across the entire human lifespan. Comprising data from 25 patients, aged between 1 day and 100 years (see Table S1), the number of labeled cells in the GCL were plotted against the age of the individual. The regression curve enables a log-log-linear interpretation. PCNA+ cell densities increase slightly with age (PPC: 0.154; p = 0.463). (0.10 MB TIF) [file pone.0008809.s005.tif]

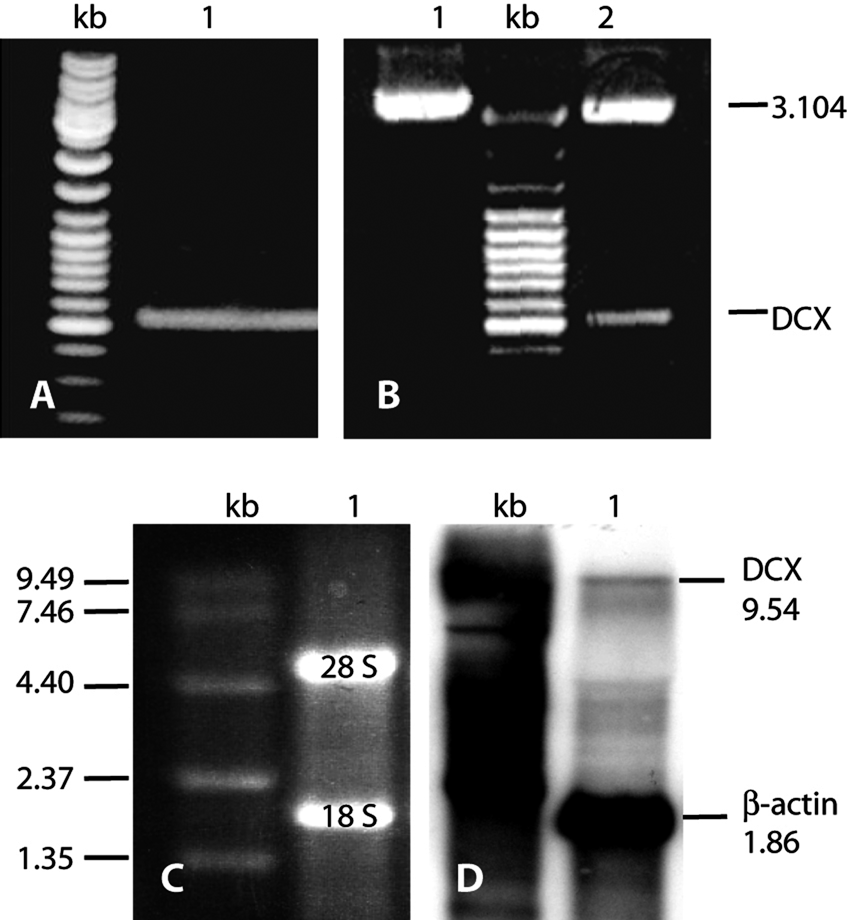

Supplement: Figure S5 — Doublecortin mRNA. Generation, purification and cloning of human DCX-specific cDNA inserts aimed to generate DIG-labeled cRNA probes for northern blotting and in situ hybridization. A, After bulk separation in a preparative 1.5% agarose gel, the fetal human brain DCX-PCR product (510 bp, lane 2) was recovered and in B, ligated into a pSPT19 vector (3.604 bp, lane 1), cloned and approved for correct insertion by digestion with EcoRI and HindIII resulting in a linearized vector (3.104 bp) and the DCX insert (lane 2) designed for the cRNA-DIG probe generation by in vitro transcription. C, Separation of total RNA from fetal brain in an ethidium bromide stained acrylamide gel (lane 1) showing strong fluorescence of the 18S and 28S ribosomal rRNAs. D, After blotting on nylon membrane the immobilized RNA was simultaneously hybridized with the DIG-labeled antisense probes against DCX and β-actin mRNAs and processed for visualization of the mRNA/cRNA-DIG binding sites by the enhanced chemiluminescence method. Besides the very strong signal of β-actin mRNA serving as housekeeping transcript and loading control a faint band of DCX mRNA was visible at the correct position of 9.54 kb. (0.26 MB TIF) [file pone.0008809.s006.tif]
