# Supplementary material for: Murine Features of Neurogenesis in the Human Hippocampus across the Lifespan from 0 to 100 Years
Source: PLoS One. 2010 Jan 29;5(1):e8809. doi: 10.1371/journal.pone.0008809 (PMC2813284; doi:10.1371/journal.pone.0008809)
Supplement: Table S1 — Analysis of perimortal hypoxic changes. (0.04 MB DOC) [file pone.0008809.s007.doc]

**Table S1** Analysis of perimortal hypoxic changes

|  | **2 mo** | **17 y** | **33 y** | **38 y** | **58 y** | **75 y** | **100 y** | **GBM** |
| --- | --- | --- | --- | --- | --- | --- | --- | --- |
| PCNA | x |  |  |  | x |  |  | x |
| Casp.-3 | x |  | x |  | x |  |  | x |
| MMP-9 |  |  | x |  |  |  | x | x |
| VEGF-A | x |  | x |  | x |  | x | x |
| Glut1 |  |  |  |  |  | x |  | x |
| HIF-1a |  |  | x |  |  | x | x | x |
| CD68 |  | x | x | x |  | x | x |  |
| HSP27 |  | x |  |  |  | x | x | x |

A sample of glioblastoma multiforme (GBM) was used as positive control.
