# Supplementary material for: Murine Features of Neurogenesis in the Human Hippocampus across the Lifespan from 0 to 100 Years
Source: PLoS One. 2010 Jan 29;5(1):e8809. doi: 10.1371/journal.pone.0008809 (PMC2813284; doi:10.1371/journal.pone.0008809)
Supplement: Table S2 — Data of subjects included in the study. (0.13 MB DOC) [file pone.0008809.s008.doc]

**Table S2** Data of subjects included in the study

| **Pat.-No.** | **Gender** | **Age (years)** | **Age (days)** | **DCX+ cells/ mm²** | **PCNA+ cells/ mm²** | **PMI**  **(h)** | **Analyses performed** | **Cause of death** |
| --- | --- | --- | --- | --- | --- | --- | --- | --- |
| 1 |  |  | GW11 | n.d. | n.d. | 1 | IHC, ISH | elective abortion |
| 2 |  |  | GW20 | n.d. | n.d. | 20 | WB | elective abortion |
| 3 | f |  | GW40 | n.d. | n.d. | 24 | ISH | stillborn child |
| 4 | f | 0.00274 | 1 | 186 | 14 | 18 | CC | stillborn child |
| 5 | m | 0.00548 | 2 | 220 | 76 | 24 | CC | respiratory insufficiency |
| 6 | m | 0.02466 | 9 | 108 | 28 | 36 | CC,IHC | stillborn child |
| 7 | m | 0.12603 | 46 | 80 | 26 | 12 | CC | respiratory insufficiency |
| 8 | f | 0.13425 | 49 | 173 | 34 | 30 | CC | cardiac insufficiency |
| 9 | m | 0.15342 | 56 | 103 | n.d. | 12 | CC,IHC | cardiac insufficiency |
| 10 | f | 0.61370 | 224 | 83 | n.d. | 42 | CC | respiratory insufficiency |
| 11 | f | 0.79452 | 290 | 81 | 63 | 60 | CC | respiratory insufficiency |
| 12 | m | 1.21370 | 443 | 23 | 17 | 30 | CC | respiratory insufficiency |
| 13 | m | 1.39726 | 510 | 23 | 2 | 24 | CC | respiratory insufficiency |
| 14 | m | 2.25 | 820 | 5 | n.d. | 30 | CC | cardiac insufficiency |
| 15 | m | 3.07 | 1.119 | 8 | 48 | 36 | CC, WB | multiple organ failure |
| 16 | f | 3.78 | 1.381 | 11 | n.d. | 8 | IHC | septic shock |
| 18 | f | 5.14 | 1.877 | 4 | n.d. | 60 | CC | multiple organ failure |
| 19 | f | 5.16 | 1.885 | 12 | n.d. | 12 | CC,ISH | cardiac insufficiency |
| 20 | m | 6.30 | 2.299 | 10 | 55 | 6 | CC,IHC | respiratory insufficiency |
| 21 | m | 11.72 | 4.279 | 4 | n.d. | 20 | CC | multiple organ failure |
| 22 | f | 12.96 | 4.731 | 5 | 67 | 24 | CC | respiratory insufficiency |
| 23 | f | 15.61 | 5.696 | 3 | n.d. | 20 | CC,CC | Hurst’s disease (AHEM) |
| 24 | m | 16.15 | 5.895 | 6 | 87 | 24 | CC,IHC | respiratory insufficiency |
| 25 | m | 16.38 | 5.979 | 5 | 53 | 36 | CC | hypovolaemic shock |
| 26 | f | 18.24 | 6.656 | 4 | n.d. | 48 | CC | toxic shock |
| 27 | m | 19.18 | 7.001 | 5 | 104 | 16 | CC | multiple organ failure |
| 28 | f | 19.31 | 7.049 | 4 | n.d. | 20 | CC | respiratory insufficiency |
| 29 | m | 20.11 | 7.339 | 6 | 24 | 24 | CC | respiratory insufficiency |
| 30 | f | 22.94 | 8.374 | 2 | n.d. | 30 | CC | cardiac insufficiency |
| 31 | f | 24.09 | 8.792 | 2 | n.d. | 18 | CC | respiratory insufficiency |
| 32 | f | 26.75 | 9.763 | 4 | n.d. | 16 | CC | haemorrhagic shock |
| 33 | m | 27.34 | 9.979 | 4 | n.d. | 16 | CC,IHC | respiratory insufficiency |
| 34 | f | 27.91 | 10.187 | n.d. | n.d. | 48 | ISH | respiratory insufficiency |
| 35 | f | 31.87 | 11.632 | 6 | n.d. | 36 | CC,CC | respiratory insufficiency |
| 37 | f | 34.76 | 12.689 | 3 | n.d. | 24 | CC,CC | multiple organ failure |
| 38 | m | 37.81 | 13.800 | 3 | n.d. | 20 | CC,CC | respiratory insufficiency |
| 39 | f | 38.79 | 14.158 | 2 | n.d. | 20 | CC,IHC | respiratory insufficiency |
| 40 | f | 40.00 | 14.600 | 1 | 28 | 16 | CC | hepatic failure |
| 41 | f | 43.00 | 15.695 | 3 | 51 | 20 | CC | hepatic and renal failure |
| 42 | m | 45.00 | 16.425 | 3 | n.d. | 36 | CC | respiratory insufficiency |
| 43 | f | 58.00 | 21.170 | 2 | 30 | 8 | CC,IHC | multiple organ failure |
| 44 | m | 62.00 | 22.630 | 1 | n.d. | 32 | CC | myocardial infarction |
| 45 | m | 65.00 | 23.725 | 2 | 99 | 30 | CC | respiratory insufficiency |
| 46 | m | 70.00 | 25.550 | 3 | 65 | 20 | CC,IHC | cardiac insufficiency |
| 47 | f | 75.00 | 27.375 | 2 | 117 | 32 | CC,IHC | septic shock |
| 48 | f | 79.00 | 28.835 | 3 | 20 | 20 | CC,IHC | cardiac insufficiency |
| 49 | m | 85.00 | 31.025 | 2 | n.d. | 30 | CC | myocardial infarction |
| 50 | f | 89.00 | 32.485 | 1 | 23 | 30 | CC | cardiac insufficiency |
| 51 | m | 94.00 | 34.310 | 1 | 17 | 48 | CC | respiratory insufficiency |
| 52 | f | 100.00 | 36.500 | n.d. | 75 | 48 | IHC | cardiac insufficiency |
| 53 | f | 60 | 21.900 | n.d. | n.d. | 1 | IHC-control | glioblastoma multiforme |
| 54 | f | 38 | 13.870 | n.d. | n.d. | 1 | WB-control | temporal lobe epilepsy |

CC = cell counting

IHC = confocal immunohistochemistry

ISH = *in situ* hybridization

PMI = postmortem intervall (h)

WB = western blot analysis

n.d. = not determined
