# Supplementary material for: Murine Features of Neurogenesis in the Human Hippocampus across the Lifespan from 0 to 100 Years
Source: PLoS One. 2010 Jan 29;5(1):e8809. doi: 10.1371/journal.pone.0008809 (PMC2813284; doi:10.1371/journal.pone.0008809)
Supplement: Table S3 — Quantification of PCNA and GFAP co-expressing undifferentiated cells in the dentate gyrus. (0.05 MB DOC) [file pone.0008809.s009.doc]

**Table S3** Quantification of PCNA and GFAP co-expressing undifferentiated cells in the dentate gyrus

| **Pat.-No.** | **Gender** | **Age (years)** | **Age (days)** | **PCNA+ cells per GCL profile** | **PCNA+/GFAP+**  **colocalizations** |
| --- | --- | --- | --- | --- | --- |
| 4 | m | 0.00548 | 2 | 7 | 0 |
| 5 | m | 0.02466 | 9 | 4 | 4 |
| 7 |  | 0.13425 | 49 | 7 | 3 |
| 8 | m | 0.15342 | 56 | 3 | 0 |
| 10 | f | 0.79452 | 290 | 2 | 0 |
| 16 | f | 5.14 | 1.877 | 1 | 0 |
| 17 | f | 5.16 | 1.885 | 2 | 0 |
| 25 | m | 19.18 | 7.001 | 1 | 0 |
| 29 | f | 24.09 | 8.792 | 4 | 0 |
| 30 | f | 26.75 | 9.763 | 12 | 3 |
| 34 | f | 34.76 | 12.689 | 6 | 1 |
| 38 | f | 43.00 | 15.695 | 16 | 2 |
| 42 | m | 65.00 | 23.725 | 2 | 0 |
| 48 | m | 94.00 | 34.310 | 1 | 0 |
| Total |  |  |  | 68 | 13 |
| Ratio |  |  |  | 100% | 19% |
