# Supplementary material for: Murine Features of Neurogenesis in the Human Hippocampus across the Lifespan from 0 to 100 Years
Source: PLoS One. 2010 Jan 29;5(1):e8809. doi: 10.1371/journal.pone.0008809 (PMC2813284; doi:10.1371/journal.pone.0008809)
Supplement: Table S4 — List of primary antibodies. (0.10 MB DOC) [file pone.0008809.s010.doc]

**Table S4** List of primary antibodies

| **Antigen** | **Chemical character; Synonym** | **Physiological character** | **Host** | **Species specificity** | **Supplier or manufacturer** | **Lot No. or**  **clone No.** | **Dilution factor** |
| --- | --- | --- | --- | --- | --- | --- | --- |
| Calretinin | calcium-binding protein | immature neuronal marker | mouse | human | Transduction Laboratories | C69520 | 250 |
| Caspase-3, activated | cysteine aspartic acid protease | apoptosis-related marker | rabbit | human | R&D Systems | AF835 | 1000 |
| Doublecortin = DCX | „Double cortex“- syndrom associated | transiently expressed in neuronal precursor cells | Goat | human, mouse… | Santa Cruz | sc-8066 | 200 |
| GABA ARα6 | GABAA receptor, subunit α6 | GABAergig cell marker | rabbit | human, mouse… | Chemicon | AB5610 | 1000 |
| GAPDH | [Glycolyse](http://de.wikipedia.org/wiki/Glycolyse) enzyme | house keeping protein | mouse | human, mouse… | Chemicon | MAB374 | 3000 |
| GAD65/67 | glutamate decarboxylase | GABAergic cell marker | rabbit | human, mouse… | Chemicon | AB1511 | 200 |
| GFAP | glial fibrillary acidic protein | astrocyte marker | mouse |  | BD Pharmingen | 556329 | 250 |
| GluR4 | glutamate receptor 4 | glutamatergic cell marker | mouse | human, monkey…. | Chemicon | MAB396 | 500 |
| Glut-1 | major glucose transporter | blood-brain-barrier | mouse | human | R&D Systems | MAB1418 | 20 |
| HIF-1**α** | Hypoxia-Inducible Factor-1alpha | hypoxia indicator | rabbit | human, mouse… | Santa Cruz | sc-10790 | 200 |
| HSP27 | Heat Shock Protein 27 | noxious stimuli indicator | rabbit |  | Stressgen | SPA 803 | 600 |
| Ki67 | proliferation marker | attached to the  chromosomes during M phase | mouse | human | Dianova | M 501 | 100 |
| MCM2 | minichromosome maintenance protein 2 | DNA replication marker | rabbit | human | BD Pharmingen | 559541 | 500 |
| MMP9 | matrixmetallo-protease9 | degrade type-IV collagen | mouse | human | Quartett GmbH, Berlin | C960318 | 250 |
| Nestin,  **α** human | intermediate filament protein | neuroepithelial marker | mouse | human | Chemicon | MAB5326 | 500 |
| Nestin,  Mouse-specific | intermediate filament protein | neuroepithelial marker | mouse | mouse | Chemicon | MAB353 | 500 |
| NeuN | neuron nuclear antigen | postmitotic and maturation marker | mouse | mouse, human… | Chemicon | MAB377 | 500 |
| NeuroD1 | neurogenic differentiation 1 | transcription marker | rabbit | human, mouse… | abcam | ab13563 | 200 |
| PCNA | proliferating cell nuclear antigen | DNA replication marker | rabbit | human, mouse… | abcam | ab2426 | 400 |
| Prox1 | prospero-related homeobox gene 1 | early marker of neural precursors | rabbit | human, mouse… | Chemicon | AB5475 | 500 |
| PSA-NCAM | polysialylated forms of the neural cell adhesion molecule | extracellular cell-surface marker of immature neuronal cells | mouse | human, mouse… | Chemicon | MAB5324 | 400 |
| Sox2 | SRY-related HMG-box gene 2 | maintains self-renewal of undifferentiated [stem cells](http://en.wikipedia.org/wiki/Stem_cell). | rabbit | human, mouse… | Chemicon | AB5603 | 500 |
| ß-III-tub | Tubulin isotype beta 3 | early cytoskeleton marker | mouse | human, rat… | Sigma | T8660 | 1000 |
| TUC-4  = TOAD | turned on after division | early post-mitotic neurons | rabbit | human, mouse… | Chemicon | AB5454 | 500 |
| VEGF-A | vascular endothelial growth factor A | proliferation of vascular endothelial cells | rabbit | human, mouse… | Santa Cruz | sc-152 | 200 |
