# Supplementary material for: Murine Features of Neurogenesis in the Human Hippocampus across the Lifespan from 0 to 100 Years
Source: PLoS One. 2010 Jan 29;5(1):e8809. doi: 10.1371/journal.pone.0008809 (PMC2813284; doi:10.1371/journal.pone.0008809)
Supplement: Table S5 — Fluorochrome conjugated secondary antibodies and optical/technical parameters for their detection in the Leica TCS NT confocal laser scanning microscope. (0.03 MB DOC) [file pone.0008809.s011.doc]

**Table S5** Fluorochrome conjugated secondary antibodies and optical/technical parameters for their detection in the Leica TCS NT confocal laser scanning microscope

| **Fluorochromes**  **(Molecular Probes, Eugene,OR)** | **Excitation** | **Excitation beam splitter** | **Detection beam splitter1** | **Suppression filter** | **Working**  **concentration**  **(µg/ml)** |
| --- | --- | --- | --- | --- | --- |
| DAG Alexa-488 | 488 nm | TK 510 | mirror | LP515 | 2 |
| DAM Alexa-555 | 568 nm | DD 488/568 | empty | LP590 | 2 |
| DAR Alexa-647 | 647 nm | RSP 660 | empty | LP665 | 2 |
